# On the risk of using raw regional data on new HIV infections in France

**DOI:** 10.1002/jia2.26205

**Published:** 2024-01-09

**Authors:** Marc‐Florent Tassi, Karl Stéfic, Cathie Faussat, Catherine Aumond, Clément Le Roux, Guillaume Gras, Leslie Grammatico‐Guillon

**Affiliations:** ^1^ INSERM U1259 MAVIVH, Université de Tours Tours France; ^2^ Laboratoire de virologie et CNR VIH‐Laboratoire associé, CHRU de Tours Tours France; ^3^ Pôle santé publique et prévention, CHRU de Tours Tours France; ^4^ Délégation régionale AIDES Centre‐Val de Loire Tours France; ^5^ Coordination Régionale de la lutte contre le VIH, CHRU de Tours Tours France; ^6^ Centre gratuit d'information, de dépistage et de diagnostic, CHRU de Tours Tours France

1

Dear Editor,

We read with particular interest the article by Wang H et al., analysing the spatio‐temporal evolution of pre‐exposure prophylaxis (PrEP) use among French men who have sex with men (MSM) over the 2016–2021 period [1], as part of the authorship has been actively promoting and studying PrEP intake for several years in the French Centre‐Val de Loire region (CVL): implementation of teleconsultations dedicated to PrEP, involvement of community players in these consultations and targeted prevention initiatives [2, 3, 4]. Thus, we were proud to discover that our region was ranked first in PrEP uptake among MSM, with twice the prevalence rate of other French regions. While pleased with these results, we, however, remained cautious and, as did the authors of the study, looked at the potential origins of such a large difference in estimates between French regions.

Even if part of the explanation may lie in the dynamism of the local community involved in sexual health challenges, this dynamism is not specific to the CVL region, thus probably insufficient to explain the difference in PrEP uptake estimated across the metropolitan regions.

Wang et al. applied spatio‐temporal ecological modelling to underline the geographical disparities and inequalities in PrEP uptake. In that end, they first estimated the size of the regional HIV‐negative MSM populations using notably unadjusted regional data on notifications of new HIV infections (both recent and late diagnosis) and 2017 European‐MSM‐Internet‐Survey data. There is no doubt that Bayesian modelling is of interest to produce robust inference about the variations of PrEP uptake in small populations. However, focusing on the factors that are likely to have an influence on the regional estimations, we could wonder if the old retrospective data sources allowed robust and reliable regional estimations, especially regarding the 2017 unadjusted HIV notification data.

It is important to note that the French system for reporting new HIV infections leads, for several reasons, to underestimated raw data and large fluctuations across years. Thus, the unadjusted data reporting 11 cases of infection among MSM in the CVL region in 2017 must be compared with the 30 or so cases estimated after taking into account underreporting, or with the 26 cases of infection reported locally by the regional HIV committee (COREVIH‐CVL). Based on these raw 2017 data, the CVL region would have the lowest estimated proportion of HIV‐negative MSM among adult men (0.3%) in France (1.4%), giving a major impact on the regional estimation of PrEP uptake. As Bayesian inference assumes prior beliefs, our own belief is that it is highly unlikely that the proportion of MSM among adult men in the CVL region would be half that of other rural regions of France. We wanted to emphasize the need to discuss results with experts of the field in a translational research approach.

Finally, these results and methodological framework are of great interest but must be reviewed with caution according to the data sources. Future use of clinical data warehouses currently under development across the country, crossed with healthcare insurance data [5], could improve HIV epidemiological indicators and strengthen ecological models.

## COMPETING INTERESTS

The authors have no relevant financial or non‐financial interests to disclose.

## AUTHORS’ CONTRIBUTIONS

M‐FT, KS and LG‐G wrote the first draft of the manuscript. All authors commented on previous versions of the manuscript. All authors read and approved the final manuscript.

## Data Availability

The authors declare that no funds, grants or other support were received during the preparation of this manuscript.
